# Impacts of single nucleotide polymorphisms in three microRNAs (miR-146a, miR-196a2 and miR-499) on the susceptibility to cervical cancer among Indian women

**DOI:** 10.1042/BSR20180723

**Published:** 2019-04-16

**Authors:** Nisha Thakur, Pallavi Singhal, Ravi Mehrotra, Mausumi Bharadwaj

**Affiliations:** 1 Division of Molecular Genetics and Biochemistry, National Institute of Cancer Prevention and Research (ICMR), Noida, Uttar Pradesh, India; 2 Division of Preventive Oncology, National Institute of Cancer Prevention and Research (Formerly Institute of Cytology and Preventive Oncology (ICPO) (ICMR), I-7, Sector-39, Noida, Gautam Buddha Nagar, Uttar Pradesh 201301, India

**Keywords:** biomarkers, cervical cancer, India, microRNA, SNP, single nucleotide polymorphisms

## Abstract

**Background:** Cervical cancer is the second major female cancer in India and constitutes one-fourth of the world’s burden. Human Papilloma Virus (HPV) infection is an essential but insufficient cause for cervical cancer. Genetic variants in microRNAs (miRNAs/miRs) play an important role in the susceptibility of various types of cancers.

**Objective:** To evaluate the association of Single Nucleotide Polymorphisms (SNPs) in miR-146a (rs2910164), miR-196a2 (rs11614913), and miR-499 (rs3746444), with cervical cancer susceptibility in Indian population.

**Methods:** Three hundred samples were genotyped by Polymerase chain reaction (PCR)-Restriction fragment length polymorphism (RFLP). Both patients and controls were also screened for the presence of HPV DNA.

**Results:** In this case–control study, 125 (83.3%) cervical cancer cases were found to be infected with HPV DNA. The frequency of miR-146a C allele was higher in controls than in cases [odds ratio (OR) (95% confidence interval (CI)) = 0.81 (0.57–1.14), *P*-value = 0.258]. miR-196a2 T allele was found to be associated with the decreased risk of cervical cancer [OR (95% CI) = 0.36 (0.26–0.50), *P*-value<0.0001]. Approximately 1.22-fold increased risk has been observed in individuals carrying miR-499 TT genotypes [OR (95% CI) = 1.22 (0.63–2.36), *P*-value = 0.617]. Interaction studies for miR-196a2/miR-499 loci showed that women carrying TT/CC and TT/CT genotypes were less likely to develop cervical cancer than CC/CC combination [*P*<0.05]. Likewise, miR-146a/miR-196a2 genotypic combinations (CC/TT, CG/TT, GG/TT) followed the similar trend [*P*<0.05], exhibited the protective effect against cervical cancer with reference to CC/CC group. Combined genotypes of miR-146a/miR-499 [CC/CT, CG/CC, CG/CT, CG/TT, GG/CC, GG/CT, GG/TT] demonstrated a non-significant trend toward higher cervical cancer risk [OR > 1.00, *P*>0.05].

**Conclusion:** Polymorphisms in miR-146a, miR-196a2, and miR-499 individually or collectively have the prospective to emerge as biomarkers for cervical cancer.

## Introduction

Cervical cancer is the third most common cancer among women globally, with an estimated 569,847 new cases and 311,365 deaths in 2018. In India, approximately 96,922 new cervical cancer cases are diagnosed annually (estimates for 2018) [1]. It lines second after breast cancer among Indian women [2]. It has been widely demonstrated that persistent infection with oncogenic high-risk human papilloma virus (HR-HPV) is the requisite for the development of cervical cancer and more than half of the cases are associated with HPV-16 [3–5].

miRNAs were first discovered in *Caenorhabditis elegans* [6]. miRNAs are small non-coding RNAs that negatively regulate gene expression in a sequence-specific manner. miRNAs are single-stranded RNAs of approximately 22 nucleotides in length that are generated by the Ribonulease III (RNase-III) type enzyme. Post-transcriptional silencing of target genes by miRNA occurs either by specific cleavage of homologous mRNA or by specific inhibition of protein synthesis. miRNAs are essential regulators of various processes such as proliferation, differentiation, development, apoptosis, and host–virus interaction. These extremely conserved RNAs control gene expression by specific inhibition of translation, induction of mRNA cleavage and DNA methylation. Approximately 1–5% of predicted genes in animals encode miRNAs and 10–30% of protein-coding genes are predicted targets regulated by miRNAs. A significant development to understand the complex behavior of the tumor cells has been possible only after exploring the numerous genes producing non-coding RNAs [7]. miRNAs are considered imperative ensemble governing various cellular phenomenon such as cell cycle regulation, programmed cell death, metastasis etc. Hence, miRNAs have been considered to also encompass the etiology, development, and prognosis of several diseases including cancer [8]. Previous studies have revealed the alterations in miRNA pattern and their role in tumor development [9–11]. Single nucleotide polymorphisms (SNPs), mutations, altered expression or processing of mature miRNA are expected to exert many phenotypic changes and may affect the susceptibility and progression of cancer. Unlike protein-coding genes, SNPs within the functional seed sequences of miRNAs are rare, occurring in *<*1% of miRNAs [12]. In dbSNP database, over 400 miRNAs SNPs have been predicted [13]. miR-146a (rs2910164), miR-196 (rs11614913), and miR-499 (rs3746444) SNPs have been reported as a key factor for carcinogenesis due to their targeting on several vital genes [14]. For the past several years, emerging molecular epidemiological studies investigated the association of these three functional SNPs and susceptibility of various cancers in different populations [15]. Functional miRNA SNP rs2910164 is located in the 3p strand of miR-146a. This polymorphism involves a mispairing in the hairpin of the precursor, which leads to altered processing, lower expression of the mature sequence [16]. This SNP (rs2910164) in the *miR-146a* locus has shown associations with lung [17], breast [18–21], prostate [22], hepatocellular carcinoma [23], esophageal [24,25], colorectal [26], gastric [27,28], glioma [29], cervical cancer [30–32], head and neck cancer [13], liver [31,33,34], gall bladder cancer [35], bladder cancer [36], thyroid cancer [16], and ovarian cancer [20].

The miR-196a2 is located on the human chromosome 12q13.13. The expression of miR-196a2 reportedly correlates with multiple kinds of malignant tumor. Accordingly, the functional variant (rs11614913) is a candidate biomarker that may influence the tumor risk [37]. Risk association with various diseases including NSCLC [38,17], familial breast cancer [15,21,39], head and neck squamous carcinoma [40], glioma [41], hepatocellular cancer [42], lung cancer [43], gastric cancer [37], renal cell cancer [44], bladder cancer [45] has been studied. SNP rs11614913 in miR-196a2 are shown to be associated with increased/decreased cancer risk. Recently, several reports identified genetic variants in the precursor or mature miRNA sequence of miR-196a2 (rs11614913, C/T) as possible biomarkers, which were associated with multiple kinds of malignant tumors, such as those that occur in the central nervous system, head and neck [40], lung [43], glioma [41], esophageal cell carcinoma [46], congenital heart disease [47], hepatocellular carcinoma [42], stomach, biliary tract, liver, breast [18], ovarian, prostate, and thyroid [48].

The *miR-499* gene is mapped on chromosome 20q11.22 and showed to be restricted within the 20th intron of the β-myosin heavy chain 7B (*Myh7b*) gene. Previous studies have reported the possible association of miR-499 with heart diseases and carcinogenesis. It also exhibited high expression in serum and were also associated with survival in non-small-cell lung cancer. Other studies have established that the genetic variant in pre-miRNA may contribute to the process of carcinogenesis in breast cancer [18], gastric cancer, and head and neck cancer. Recently it has been demonstrated that polymorphism of miR-499 is associated with oral cancer [13]. However, few reports have been published with reference to miR-499 polymorphism and cervical cancer risk [31,32], but its effect on Indian cervical cancer patients is not completely understood. To the best of our knowledge, only single report by Srivastava et al. (2017) [32] is available till date, with respect to genetic variants in miR-146a, miR-196a2, and miR-499 SNPs and cervical cancer risk in Indian population. Therefore, the present study is designed to further explore whether three functional SNPs in miR-146a (rs2910164), miR-196a2 (rs11614913), and miR-499 (rs3746444) are associated with cervical cancer susceptibility in Indian population individually or in combination.

## Materials and methods

### Experimental subjects

The present case–control study comprised 150 histologically confirmed cervical cancer cases from the Lok Nayak Jai Prakash Hospital, New Delhi. The age and ethinicity matched control group consisted of 150 women with no history of cancer. Written informed consent was taken from all the participants enrolled in the study. The study was approved by the Ethics Committee of the Institute and carried out in compliance with the principles of Helsinki Declaration. The mean age of the patients was 48.3 ± 11.5 years and that of controls was 48.0 ± 10.2 years.

### DNA isolation and HPV detection

DNA was isolated from cervical tissue biopsies and cervical scrapes samples from cases and controls, using Proteinase K method followed by phenol/chloroform/isopropanol extraction [49]. PCR for β-globin was performed to check the quality of the extracted genomic DNA by amplifying a 268-bp fragment [50]. Detection of HPV infection was done by conventional PCR using MY09 and MY11 consensus primers [51] and further HPV typing was made by PCR with type-specific primers (HPV-16/18) [52].

### Determination of microRNA genotypes

The biological characteristics for miR SNPs under investigation have been extracted from dbSNP (http://www.ncbi.nlm.nih.gov/SNP) and summarized in Table 1. The secondary structures of respective wild-type and polymorphic microRNAs have been searched in database for microRNA-related SNPs (http://www.bioguo.org/miRNASNP) as shown in Supplementary Figures S1–S3). PCR-Restriction fragment length polymorphism (RFLP) method was employed to determine the single nucleotide variation of miR-146a (rs2910164), miR-196a2 (rs11614913), and miR-499 (rs3746444) as previously described [17].

**Table 1:** Characteristics of three miR SNPs investigated in the present study

Gene ID	Organism	Molecule type	NCBI dbSNP ID	Location	Chromosome (position)	Alleles	Ancestral allele	Global MAF/minor allele count	Functional consequences
miR-146a	*Homo sapiens*	Genomic	rs2910164	Pre-miRNA	5 (160485411)	C/G	G	C = 0.2792/32442 (ExAC)^2^ C = 0.2797/2881 (GO-ESP)^3^	nc^4^ transcript variant
miR-196a2	*Homo sapiens*	Genomic	rs11614913	Pre-miRNA	12 (53991815)	C/T	C	T = 0.3327/1666 (1000 Genomes)^1^	nc^4^ transcript variant
miR-499	Homo sapiens	Genomic	rs3746444	pre-miRNA	20 (34990448)	C/T	T	G = 0.1835/919 (1000 Genomes)^1^	nc^4^ transcript variant, intron variant

Abbreviation: MAF, minor allele frequency. *Source:* NCBI (http://www.ncbi.nlm.nih.gov) and

1 1000Genome (http://www.1000genomes.org)

2 Exome Aggregation Consortium (ExAC) (http://exac.broadinstitute.org)

3 NHLBI GO Exome Sequencing Project (ESP) (https://esp.gs.washington.edu)

4 nc, non-coding.

### miR-146a polymorphism (rs2910164)

Briefly, for genotyping of miR-146a G/C SNP (rs2910164), a 147-bp fragment was amplified using forward 5-CATGGGTTGTGTCAGTGTCAGAGCT-3 and reverse 5-TGCCTTCTGTCTCCAGTCTTCCAA-3 primers. The amplified PCR product (Supplementary Figure S4a) was digested overnight at 37°C with two units of *SacI* restriction enzyme. Ten percent polyacrylamide gel electrophoresis (PAGE) was used to study the genotypic pattern of the digested PCR products (Supplementary Figure S4b,c). A single fragment of 147 bp shows G allele (wild-type), whereas the fragments of 122 and 25 bp confirmed the presence of variant C allele.

### miR-196a2 polymorphism (rs11614913)

Genotyping of miR-196a2 C/T SNP (rs11614913) was performed by using forward 5-CCCCTTCCCTTCTCCTCCAGATA-3 and reverse 5-CGAAAACCGACTGATGTAACTCCG-3 primer set. The two units of *MspI* restriction enzyme was used to digest 149-bp PCR product (Supplementary Figure S5a) overnight at 37°C. The digested PCR products were checked on 10% PAGE (Supplementary Figure S5b). Two fragments of 125 and 24 bp indicate the presence of C allele (wild-type), and the single fragment of 149 bp shows T allele (variant).

### miR-499 polymorphism (rs3746444)

To genotype miR-499 C/T SNP (rs3746444), a 146-bp fragment was generated by using forward 5-CAAAGTCTTCACTTCCCTGCCA-3 and reverse 5-GATGTTTAACTCCTCTCCACGTGATC-3 primers. The amplified PCR product of 146 bp (Supplementary Figure 6a) was digested overnight at 37°C with two units of *BclI* restriction enzyme. The digested PCR products were resolved on 10% PAGE (Supplementary Figure S6b). The single fragment of 146 bp showed the C allele (wild-type), and the variant T allele produced two fragments of 120 and 26 bp.

### Analysis

The power of the study was >80% calculated by QUANTO software (http://biostats.usc.edu/cgi-bin/DownloadQuanto.pl). The odds ratio (OR) and its 95% confidence intervals (CI) were also calculated to determine the relationship between the miR genotypes and cervical cancer risk. The significance of statistical test Chi-square/Fisher’s exact was measured as two-tailed. The *P*-value of <0.05 was considered statistically significant. The genotypic and allelic frequencies were further checked for the conformance of Hardy–Weinberg equilibrium (HWE). The complete statistical analyses were performed using GraphPad Instat V.3.1.

## Results

### HPV status

Out of 150 cervical cancer cases, 125 (83.3%) were found to be infected with HPV by PCR using MY09/MY11 L1 consensus primers. On further high-risk HPV genotyping by HPV type specific PCR we observed that 123 (98.4%) cases were positive for HPV type-16 and 2 (1.6%) were infected with HPV type-18 out of total HPV positive cases. Whereas, only six (4%) samples from healthy control group showed the presence of HPV DNA sequences and all of them were infected with HPV type-16.

### miR polymorphisms

The genotype and allele frequencies of miR-146a, miR-196a2, and miR-499 polymorphisms were compared between controls and cervical cancer patients (Table 2). All the three miR genotypic and allelic distributions studied in the present study were in HWE.

**Table 2:** Distribution of miRNA genotypic and allelic frequencies in cervical cancer cases and controls

	Control (*n*=150)	Cases (*n*=150)	*P*-value	OR (95% CI)
	*n* (%)	*n* (%)		
**miR-146a (rs2910164)**				
**Genotype frequency**				
GG	73 (48.7)	80 (53.3)		1.00 (Reference)
GC	49 (32.7)	49 (32.7)	0.823	0.91 (0.54–1.52)
CC	28 (18.6)	21 (14.0)	0.325	0.68 (0.35–1.31)
GC+CC	77 (51.3)	70 (46.7)	0.488	0.83 (0.53–1.31)
**Allelic frequency**				
G	195 (65)	209 (69.7)		1.00 (Reference)
C	105 (35)	91 (30.3)	0.258	0.81 (0.57–1.14)
				
**miR-196 (rs11614913)**				
**Genotype frequency**				
CC	42 (28)	75 (50)		1.00 (Reference)
CT	51 (34)	58 (38.7)	0.127	0.64 (0.37–1.08)
TT	57 (38)	17 (11.3)	<0.0001*	**0.17 (0.08–0.32)**
CT+TT	108 (72)	75 (50)	0.0002*	**0.39 (0.24–0.63)**
**Allelic frequency**				
C	135 (45)	208 (69.3)		1.00 (Reference)
T	165 (55)	91 (30.7)	<0.0001*	**0.36 (0.26–0.50)**
**miR-499 (rs3746444)**				
**Genotype frequency**				
CC	80 (53.3)	78 (52.0)		1.00 (Reference)
CT	49 (32.7)	47 (31.3)	0.949	0.98 (0.59–1.63)
TT	21 (14.0)	25 (16.7)	0.617	1.22 (0.63–2.36)
CT+TT	70 (46.7)	72 (48.0)	0.909	1.06 (0.67–1.66)
**Allelic frequency**				
C	209 (69.7)	203 (67.7)		1.00 (Reference)
T	91 (30.3)	97 (32.3)	0.659	1.09 (0.77–1.55)

*P*-value, probability from chi-square test comparing the genotypic/allelic distribution in cervical cancer cases and controls. Significant *P*-values are shown in bold.

### miR-146a SNP (rs2910164)

The genotypic and allelic distribution of miR-146a G/C polymorphism (rs2910164) in cervical cancer patients and healthy controls is presented in Table 2. In the present study, we found that the frequency of wild homozygous miR-146a genotype (GG) was slightly higher in cases 53.3% (80/150) as compared with controls 48.7% (73/150). It was interesting to note, that the frequency of the heterozygous genotype (GC) was identical 32.7% (49/150) both in case and control groups. The frequency of carrier genotype (GC+CC) was found to be considerably higher in controls 51.3% (77/150) than in cases 46.7% (70/150) but this difference was statistically not significant [*P*=0.488; OR (95% CI) = 0.83 (0.53–1.31)]. The polymorphic homozygous genotype (CC) was remarkably higher in controls 18.6% (28/150) in comparison with cases 14.0% (21/150) but this difference could not reach the limits of statistical significance [*P*=0.325; OR (95% CI) = 0.68 (0.35–1.31)]. Additionally, it was reported that the frequency of miR-146a polymorphic C allele was higher in controls 35% (105/300) in comparison with cases 30.3% (91/300) but this difference was also not statistically significant [*P*=0.258; OR (95% CI) = 0.81 (0.57–1.14)] (Table 2). This finding indicates lack of association between miR-146a SNP (rs2910164) and cervical cancer risk in Indian population.

### miR-196a2 SNP (rs11614913)

The genotypic and allelic distribution of miR-196a2 polymorphism C/T (rs1161493) in cervical cancer patients and healthy controls is presented in Table 2. We observed that the frequency of wild-type homozygous miR-196a2 genotype (CC) was higher in cases 50% (75/150) as compared with control group 28% (42/150). The occurrence of miR-196a2 heterozygous genotype (CT) was slightly higher in cases 38.7% (58/150) than in controls 34% (51/150) but this difference could not attain the limits of statistical significance [*P*-value = 0.127; OR (95% CI) = 0.64 (0.37–1.08)]. However, the frequency of miR-196a2 polymorphic homozygous genotypes (TT) was significantly higher in controls 38% (57/150) than in cases 11.3% (17/150) [*P*-value<0.0001; OR (95% CI) = 0.17 (0.08–0.32)]. It was also interesting to observe that the frequency of miR-196a2 carrier genotypes (CT+TT) was higher in controls 72% (108/150) than in cases 50% (75/150) and this difference was statistically extremely significant [*P*<0.0002; OR (95% CI) = 0.39 (0.26–0.50)]. So, we can infer from this result that the Indian women carrying miR-196a2 (CT+TT) genotypes were less likely to develop cervical cancer. Similarly, miR-196a2 polymorphic T allele was found to be associated with the decreased risk of cervical cancer [*P*-value<0.0001; OR (95% CI) = 0.36 (0.26–0.50)]. These results suggest that the SNP (rs1161493) located on miR-196a2 may play a protective role against the development of cervical cancer in Indian population.

### miR-499 SNP (rs3746444)

The genotypic and allelic distribution of miR-499 polymorphism (rs3746444) in cervical cancer cases and controls is presented in Table 2. It was interesting to note, that there was not much difference in the distribution of different genotypes of SNP rs3746444 in miR-499 between controls and cases. However, elevated frequency of miR-499 variant genotype (TT) was reported in cases 16.7% (25/150) than in controls 14% (21/150) to some extent. Women carrying miR-499 TT genotypes in relation to CC genotypes had ∼1.22-fold increased risk for the development of cervical cancer in Indian population but this difference was not statistically significant (*P*-value>0.05). The frequency of miR-499 carrier genotypes (CT+TT) was slightly higher in cases 48% (72/150) in relation to controls 46.7% (70/150) but this difference was not statistically significant [*P*-value = 0.909; OR (95% CI) = 1.1 (0.67–1.66)]. In contrary to this, lower frequency of heterozygous genotype (CT) was observed in cases 31.3% (47/150) than in controls 32.7% (49/150). However, almost equal frequency of wild-type homozygous genotype (CC) was noticed in controls 53.3% (80/150) and in cases 52% (78/150).

### Gene–Gene interaction study/combination analysis of three miR-SNPs

#### [miR-146a (rs2910164), miR-196a2 (rs11614913), and miR-499 SNP (rs3746444)]

Combined analysis of different genotypes of three miR-SNPs is presented in Table 3. We made three different combinations as [miR-146a/miR-196a2], [miR-146a/miR-499], and [miR-196a2/miR-499] to study the interaction of various miR-genotypes and their effect on cervical cancer risk.

**Table 3:** Combination analysis of miR-146 G/C, miR-196a2 C/T, and miR-499 C/T polymorphisms in Indian cervical cancer cases and controls

Genotype	Controls	Cases	*P*-value	OR (95% CI)
***miR-146a/miR-196a2***				
CC/CC	7 (4.67)	13 (8.67)		1.000 (Reference)
CC/CT	9 (6)	8 (5.33)	0.3309	0.4786 (0.1274–1.798)
CC/TT	12 (8)	0 (0)^1^	**0.0004**	**0.02222 (0.001146–0.4310)**
CG/CC	17 (11.33)	27 (18)	1	0.8552 (0.2843–2.573)
CG/CT	13 (8.67)	13 (8.67)	0.3767	0.5385 (0.1625–1.785)
CG/TT	20 (13.33)	9 (6)	**0.0234**	**0.2423 (0.07226–0.8125)**
GG/CC	18 (12)	35 (23.33)	1	1.047 (0.3553–3.085)
GG/CT	29 (19.33)	37 (24.67)	0.607	0.6870 (0.2429–1.943)
GG/TT	25 (16.67)	8 (5.33)	**0.0045**	**0.1723 (0.05107–0.5814)**
***miR-146a/miR-499***				
CC/CC	19 (12.7)	14 (9.33)		1.000 (Reference)
CC/CT	5 (3.33)	6 (4)	0.5093	1.629 (0.4125–6.430)
CC/TT	4 (2.67)	1 (0.67)	0.6295	0.3393 (0.03408–3.377)
CG/CC	30 (20)	29 (19.33)	0.6636	1.312 (0.5558–3.096)
CG/CT	13 (8.67)	12 (8)	0.7913	1.253 (0.4404–3.563)
CG/TT	5 (3.33)	9 (6)	0.2124	2.443 (0.6703–8.903)
GG/CC	33 (22)	34 (22.67)	0.5245	1.398 (0.6034-3.240)
GG/CT	30 (20)	30 (20)	0.5214	1.357 (0.5765–3.195)
GG/TT	11 (7.33)	15 (10)	0.2993	1.851 (0.6539–5.238)
***miR-196a2/miR-499***				
CC/CC	26 (17.33)	43 (28.67)		1.000 (Reference)
CC/CT	11 (7.33)	23 (15.33)	0.6659	1.264 (0.5306–3.012)
CC/TT	5 (3.33)	9 (6)	1	1.088 (0.3288–3.603)
CT/CC	26 (17.33)	28 (18.67)	0.2732	0.6512 (0.3161–1.341)
CT/CT	16 (10.67)	19 (12.67)	0.5266	0.7180 (0.3148–1.638)
CT/TT	9 (6)	11 (7.33)	0.6085	0.7390 (0.2700–2.023)
TT/CC	29 (19.33)	7 (4.67)	**<0.0001**	**0.1460 (0.05597–0.3806)**
TT/CT	22 (14.67)	5 (3.33)	**0.0002**	**0.1374 (0.04636–0.4073)**
TT/TT	6 (4)	5 (3.33)	0.3328	0.5039 (0.1397–1.818)

1 To make calculation possible, 0.5 was added to each value. Abbreviation: P-value, probability from chi-square test comparing the genotypic/allelic distribution in cervical cancer cases and controls. Significant *P*-values are shown in bold.

In combination analysis of the genotypes of three miR-SNPs, it was very surprising to note that the CC/TT genotypes for the miR-146a/miR-196a2 loci were absent from cervical cancer group. However, the frequency of this combination was significantly higher in control group 8% (12/150) than in cases 0% (0/150)]. To make the calculation possible, 0.5 was added to each value. Overall, three combination groups including CC/TT, CG/TT, and GG/TT genotypes were found to be significantly associated as a protective factor against the development of cervical cancer with reference to CC/CC genotypes of miR-146a/miR-196a2 loci [*P*-value = 0.0004; OR (95% CI) = 0.022 (0.0011–0.43); *P*-value = 0.0234; OR (95% CI) = 0.24 (0.07–0.81); *P*-value = 0.005; OR (95% CI) = 0.17 (0.05–0.58), respectively]. It was interesting to observe that the distribution of CG/CT genotypes was comparable with a frequency of 8.67% (13/150) among controls and cases. CC/CT genotypes also showed protection for cervical cancer up to some extent but this association was statistically not significant [*P*-value = 0.33; OR (95% CI) = 0.48 (0.13–1.79). Contrary to this, CG/CC, GG/CC, and GG/CT genotypic combination showed an inclination toward the increased risk of cervical cancer but these associations could not reach the limits of statistical significance [*P*-value = 1.000; *P*-value = 1.000; *P*-value = 0.607, respectively].

When we studied the interaction of miR-146a and miR-499 SNP, it was found that CG/TT genotypes for the miR-146a/miR-499 loci had ∼2.4-fold increased risk for cervical cancer development with reference to CC/CC genotypes but this difference was statistically not significant [*P*-value = 0.212; OR (95% CI) = 2.44 (0.67–8.9)]. CC/TT genotypes were the only group which indicated a non-significant protective role for the cervical cancer development [*P*-value = 0.63; OR (95% CI) = 0.34 (0.03–3.38)]. All the six remaining genotypic combinations including CC/CT, CG/CC, CG/CT, GG/CC, GG/CT, and GG/TT were found to be associated with ∼1.5-fold higher risk of cervical cancer independently, however, none of these groups showed a statistically significant difference when compared with the reference genotypes CC/CC of miR-146a/miR-499 loci [*P*-value = 0.509; OR (95% CI) = 1.63 (0.41–6.43); *P*-value = 0.66; OR (95% CI) = 1.31 (0.56–3.09); *P*-value = 0.791; OR (95% CI) = 1.25 (0.44–3.56); *P*-value = 0.525; OR (95% CI) = 1.39 (0.60–3.24); *P*-value = 0.521; OR (95% CI) = 1.36 (0.58–3.19), and *P*-value = 0.299; OR (95% CI) = 1.85 (0.65–5.24), respectively].

Interaction between miR-196a2 and miR-499 genetic variants showed that for miR-196a2/miR-499 loci, the TT/CC combined genotype was more frequently present in controls 19.33% (29/150) than in cases 4.67% (7/150) compared with the CC/CC genotype as the reference. So, it could be inferred from this observation that women carrying TT/CC genotypes for miR-196a2/miR-499 loci were significantly less likely to develop cervical cancer, than those carrying CC/CC genotypes [*P*-value ≤ 0.0001; OR (95% CI) = 0.15 (0.06–0.38)]. Similarly, TT/CT genotypic combination was proven as a major protective factor in relation to CC/CC genotype for miR-196a2/miR-499 loci [*P*-value = 0.0002; OR (95% CI) = 0.14 (0.05–0.41)]. Contrary to this, CC/CT and CC/TT combined genotypes were found to be associated with (1.1–1.3)-fold-non-significant increased risk of cervical cancer among Indian population with reference to CC/CC genotype for the miR-196a2/miR-499 loci [*P*-value = 0.666, OR (95% CI) = 1.26 (0.53–3.02); *P*-value = 1.000; OR (95% CI) = 1.09 (0.33–3.60)]. Remaining four genotype combinations for the miR-196a2/miR-499 loci (CT/CC, CT/CT, CT/TT, and TT/TT) were found to be non-significantly associated with the lower risk of cervical cancer in comparison with the CC/CC reference genotype [*P*-value = 0.273, OR (95% CI) = 0.65 (0.32–1.34); *P*-value = 0.527, OR (95% CI) = 0.72 (0.31–1.64); *P*-value = 0.609, OR (95% CI) = 0.74 (0.27–2.02); *P*-value = 0.333, OR (95% CI) = 0.50 (0.14–1.81), respectively].

## Discussion

It has been established that persistent infection with HPV is the primary step leading to cervical cancer, but other factors are required for the development and progression of the disease. Complex interactions between the host, viral, and environmental factors are accountable for the disease progression from precancer to cancer. Molecular alterations in oncogenes or tumor suppressor genes have been recognized as the key drivers toward cancer development. The present study provides the evidence that common SNPs in three miRNAs (miR-146a, miR-196a2, miR-499) might play a vital role for susceptibility to cervical cancer in Indian population. Very limited knowledge is available with reference to the miR-SNPs and risk of cervical cancer [53,32]. With this background, the present study was designed to focus on the influence of SNPs in miRNAs on genetic susceptibility to cervical cancer in Indian women.

A G/C polymorphism (rs2910164) is functionally significant as it is located on the passenger strand of the precursor of miR-146a, which could modify mature miR-146a expression. Studies of relationship between cancer susceptibility and miR-146a polymorphism can be traced from the time when Jazdzewski et al. in 2008 [16], first reported that the SNP (rs2910164) was associated with increased probability of acquiring papillary thyroid carcinoma. They found that the C allele of mature miR-146a would cause less-efficient inhibition of target genes including TNFR-associated factor 6 (TRAF6), IL-1R-associated kinase 1 (IRAKI1), and papillary thyroid carcinoma 1 gene (*PTC1*). SNP (G/C) in miR-146a was also found to be associated with higher risk of developing prostate cancer [22,49]. Our finding indicates a promising trend toward association of miR-146a SNP (rs2910164) with the protection of cervical cancer among Indian population. Whereas, Srivastava et al. (2017) [32], showed lack of association between miR-146a polymorphism and cervical cancer risk in India population. Contrary to this, it was found that wild- type homozygous genotype (GG) of miR-146a is associated with the risk of cervical cancer in Chinese population [30]. A significantly increased cervical squamous cell carcinoma risk was found to be associated with miR-146a G allele [31]. The current study reports a non-significant higher frequency of polymorphic miR-146a C allele in control group suggesting a potential protective role. Reports on hepatocellular and bladder cancers showed no effect of miR-146a SNP with genetic susceptibility [33,36]. Contrary to these findings breast, gastric, gall bladder and head and neck cancers were found to be linked with the increased disease risk in relation to miR-146a polymorphism [13,21,27,36,39]. miRNA profiling studies have shown that many miRNAs are up-regulated or down-regulated in different types of cancers and that most of them are down-regulated [40]. The expression of miR-196a2 reportedly correlates with multiple kinds of malignancies. Accordingly, the functional variant rs11614913 is a candidate biomarker that may influence the cancer risk [39].

The present study conducted on Indian women revealed that miR-196a2 polymorphic T allele was associated with the decreased risk for cervical cancer development. So, it could be inferred that miR-196a2 polymorphism (rs1161493) might reduce the cervical cancer risk among Indian population. However, Srivastava et al. 2017 showed no association between miR-196a2 polymorphism and cervical cancer risk in Indian population [32]. This SNP was found to be associated with significantly increased risk and poor survival among Chinese lung cancer patients [45]. However, individuals carrying the CC genotype of the miR-196a2 variant rs11614913 had increased susceptibility to lung cancer but this relationship was not true in case of hepatocellular, gastric and esophageal cancer cases [12]. SNP rs11614913 located on miR-196a2 was reported to be associated with the increased risk of breast, head & neck, gastric, renal and bladder cancer [15,40,37,44,45]. In contrast to this, miR-196a2 variant rs11614913 was found to be associated with the reduced risk of breast cancer and glioma in Chinese population [39,41]. Recent studies showed that in most of malignant tumors miR-196a2 CC/CT genotype was associated with high risk for cancer while miR-196a2 TT genotype was associated with decreased risk for cancer. Our findings are coherent with the recent published data. Thus, SNP rs11614913 in miR-196a2 may be associated with lower risk for cervical cancer.

Lack of significant association of miR-499 polymorphism (rs3746444) with the risk of cervical cancer among Indian population was observed in the present study. A meta-analysis has also been published showing no significant association in different cancers with reference to this SNP [56]. Contrary to this, miR-499 T/C polymorphism was found to be significantly associated with the risk of Indian cervical cancer patients [32]. Furthermore, a significant increased risk has been reported for the disease in Asians but not in Caucasians [54]. Our findings are also in line with the studies on breast cancer [55], bladder cancer [36], lung cancer [43], squamous cell carcinoma of head and neck [14], non-small cell lung cancer [17], gastric cancer [28] and gallbladder cancer showing no risk association in relation to miR-499 polymorphism (rs3746444). However, some reports have recommended that miR-499 polymorphism (rs3746444) was associated with the increased risk of cervical squamous cell carcinoma [31], prostate cancer [48] and oral cancer [13]. The possible explanation for such type of inconsistent results may be the small sample size, environmental factors and different ethnic background of the studied populations. Hence, it is important to consider host-genetic make-up in order to understand the mechanism of cancer susceptibility in humans.

Synergistic effect of miRNA SNPs has been studied in various disorders or pathological conditions including polycystic ovarian syndrome (PCOS), premature ovarian failure (POF) [56], oral cancer [13], prostate cancer [48] etc. Growing evidences have supported the fact that gene-gene interactions may influence the gene-disease association. However, only one report is available showing the effect of different genotypic combinations of three miR polymorphisms on the disease risk [56]. This study showed miR-146a CG/miR-196a2 TC combined genotypes as a protective factor for POF, in agreement to this, we found the similar effect of this genotypic combination with reference to cervical cancer. Our findings showed that the CC/TT, CG/TT and GG/TT combined genotypes of the miR-146a/miR-196a2 loci were significantly associated with decreased cervical cancer risk (a protective factor) with respect to CC/CC combined reference genotypes. Therefore, the present study was able to establish the gene-gene interaction and its association with cervical cancer susceptibility. Limitation of the present study was our samples were recruited from the Delhi based hospitals so these may not be the true representatives of the population under investigation.

## Conclusion

In conclusion, this case-control study provides the evidence that miR-196a2 (rs11614913) polymorphism was associated with a significantly decreased cervical cancer risk. However, miR-146a (rs2910164) and miR-499 (rs3746444) genetic variants were not associated individually, but their combinations with miR-196a2 were found to be linked with the lower cervical cancer risk in Indian population. Taken together, polymorphisms in miR-146a, miR-196a2 and miR-499 either individually or collectively have the prospect to emerge as futuristic biomarker for cervical cancer. Our understanding on the effects of miR polymorphisms with reference to the risk of cervical cancer requires validation by further larger studies.

## Supporting information

**Supplementary Figure 1:** Detail information of hsa-mir-146a SNP (rs2910164) and secondary structure (a) Sequence showing polymorphic site ‘C’ (b) Wild miR-146a (c) Polymorphic miR-146a

**Supplementary Figure 2:** Detail information of hsa-mir-196a2 SNP (rs11614913) and secondary structure (a) Sequence showing polymorphic site ‘U’ (b) Wild miR-196a2 (c) Polymorphic miR-196a2

**Supplementary Figure 3:** Detail information of hsa-mir-499 SNP (rs3746444) and secondary structure (a) Sequence showing polymorphic site ‘A’ (b) Wild miR-499 (c) Polymorphic miR-499

**Supplementary Figure 4:** **(a)** Amplification of hsa-miR-146a by PCR on 2% Agarose Gel Electrophoresis. **(b) & (c)** Analysis of hsa-miR-146a G<C polymorphism (rs2910164) by RFLP on 10% Native Polyacrylamide Gel Electrophoresis. **(b)** Lane M-φX174/*Hae* III digested Molecular Weight Marker; lanes 1,3,4- heterozygous GC; lanes 2,5,6 - homozygous CC. **(c)** lanes1,2,5,6- heterozygous GC genotype; lanes 3,8- homozygous CC genotype; lanes 4,7- homozygous GG genotype.

**Supplementary Figure 5:** **(a)** Amplification of hsa-miR-196a2 by PCR on 2% Agarose Gel Electrophoresis. **(b)** Analysis of hsa-miR-196a2 C<T polymorphism (rs11614913) by RFLP on 10% Native Polyacrylamide Gel Electrophoresis. Lane M-φX174/*Hae* III digested Molecular Weight Marker; lanes 1,3,4 & 8 - heterozygous CT genotype; lanes 2,5,6 & 7 - homozygous CC genotype; lane 9 - homozygous TT genotype.

**Supplementary Figure 6:** **(a)** hsa-miR-499 PCR Product (146bp) Lane M-φX174/*Hae* III digested Molecular Weight Marker; 1-4, Amplicon, N- Negative control; **(b)** 10% PAGE showing the RFLP of hsa-miR-499. Lane 1- homozygous genotype CC; lane 3,5- heterozygous CT genotype; lane 2,4,6- homozygous TT genotype.

## Abbreviations

CI: Confidence interval
HPV: Human papilloma virus
HWE: Hardy–Weinberg equilibrium
miRNA or miR: microRNA
OR: Odds ratio
PAGE: Polyacrylamide gel electrophoresis
POF: Premature ovarian failure
SNP: Single nucleotide polymorphism
