# Supplementary material for: Impacts of single nucleotide polymorphisms in three microRNAs (miR-146a, miR-196a2 and miR-499) on the susceptibility to cervical cancer among Indian women
Source: Biosci Rep. 2019 Apr 16;39(4):BSR20180723. doi: 10.1042/BSR20180723 (PMC6465206; doi:10.1042/BSR20180723)
Supplement: Supplementary file 1 [file bsr20180723_Supp1.pdf]

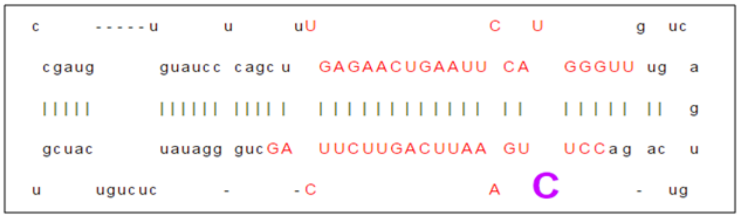

(1a)

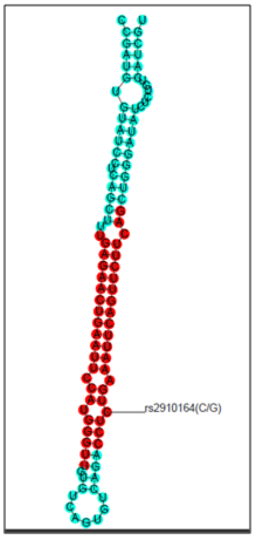

(1b)

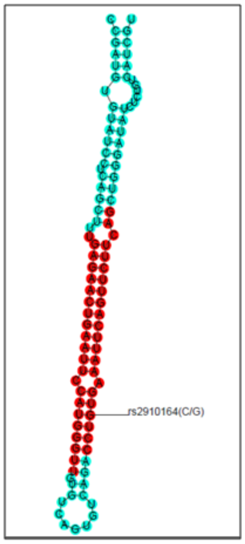

(1c)

Supplementary Figure 1. Detail information of hsa-mir-146a SNP (rs2910164) and secondary structure (a) Sequence showing polymorphic site ‘C’ (b) Wild miR-146a (c) Polymorphic miR-146a

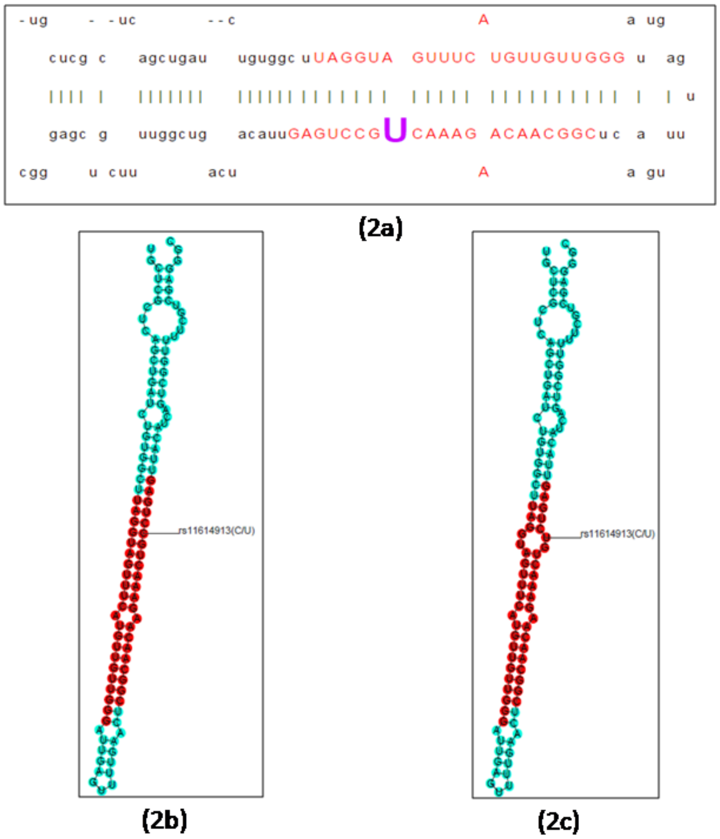

Supplementary Figure 2. Detail information of hsa-mir-196a2 SNP (rs11614913) and secondary structure (a) Sequence showing polymorphic site ‘U’ (b) Wild miR-196a2 (c) Polymorphic miR-196a2

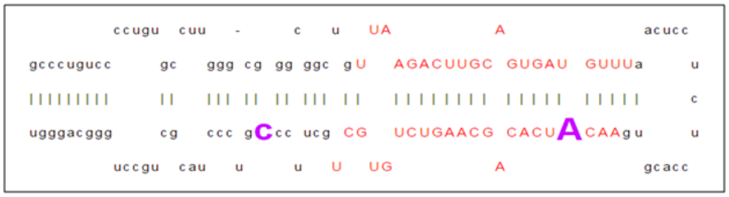

(3a)

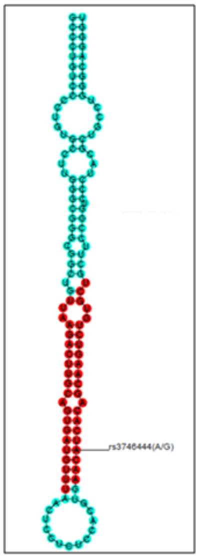

(3b)

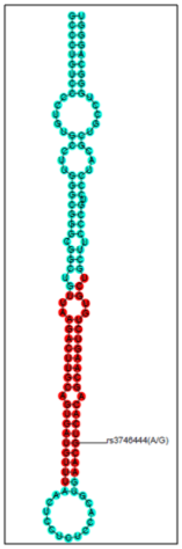

(3c)

Supplementary Figure 3. Detail information of hsa-mir-499 SNP (rs3746444) and secondary structure (a) Sequence showing polymorphic site 'A' (b) Wild miR-499 (c) Polymorphic miR-499

Note: Hairpin: mature sequence is shown in red. Note: Highlighted C in fig.(a) shows another SNP(rs7267163) which is not investigated in the present study.

Source: http://www.bioguo.org/miRNASNP/.

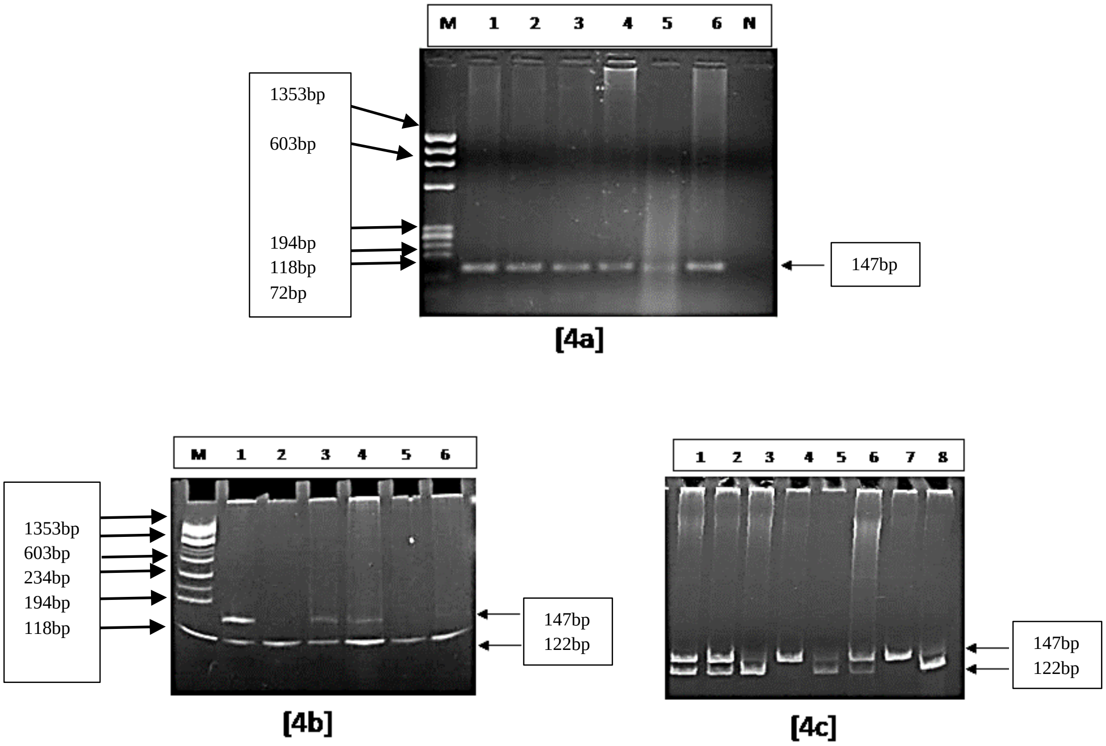

Supplementary Figure (4)(a) Amplification of hsa-miR-146a by PCR on 2% Agarose Gel Electrophoresis. **(b) & (c)** Analysis of hsa-miR-146a G<C polymorphism (rs2910164) by RFLP on 10% Native Polyacrylamide Gel Electrophoresis. **(b)** Lane M-*φ*X174/*Hae* III digested Molecular Weight Marker; lanes 1,3,4– heterozygous GC; lanes 2,5,6 – homozygous CC. **(c)** lanes1,2,5,6– heterozygous GC genotype; lanes 3,8- homozygous CC genotype; lanes 4,7- homozygous GG genotype.

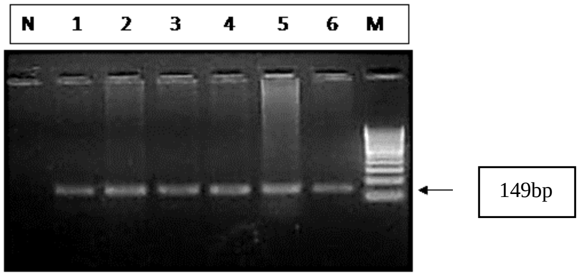

[5a]

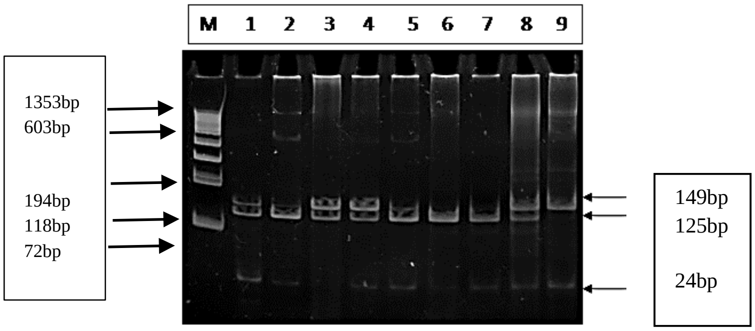

[5b]

Supplementary Figure 5(a) Amplification of hsa-miR-196a2 by PCR on 2% Agarose Gel Electrophoresis. **(b)** Analysis of hsa-miR-196a2 C<T polymorphism (rs11614913) by RFLP on 10% Native Polyacrylamide Gel Electrophoresis. Lane M-*φ*X174/*Hae* III digested Molecular Weight Marker; lanes 1,3,4 & 8 – heterozygous CT genotype; lanes 2,5,6 & 7 – homozygous CC genotype; lane 9 – homozygous TT genotype.

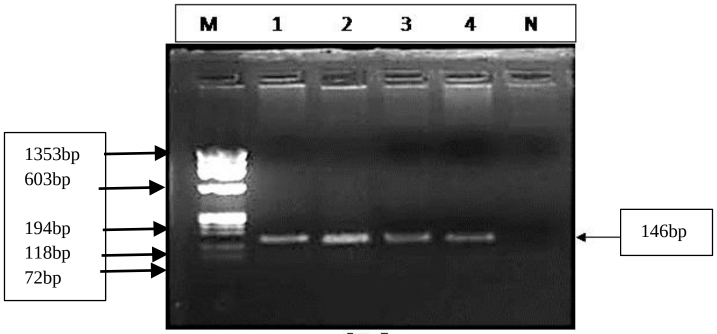

[6a]

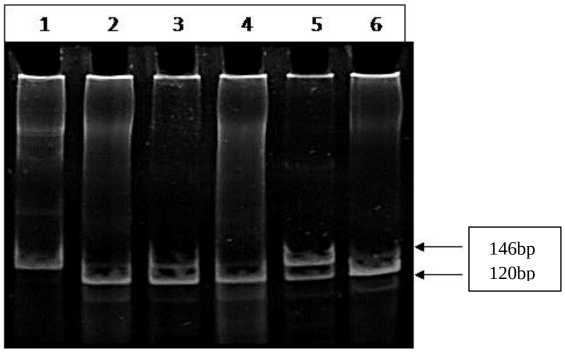

[6b]

Supplementary Figure 6(a) hsa-miR-499 PCR Product (146bp) Lane M-*φ*X174/*Hae* III digested Molecular Weight Marker; 1-4, Amplicon, N- Negative control; **(b)** 10% PAGE showing the RFLP of hsa-miR-499. Lane 1- homozygous genotype CC; lane 3,5- heterozygous CT genotype; lane 2,4,6- homozygous TT genotype.
